# Supplementary material for: One-atom-thick hexagonal boron nitride co-catalyst for enhanced oxygen evolution reactions
Source: Nat Commun. 2023 Nov 1;14:6965. doi: 10.1038/s41467-023-42696-3 (PMC10618520; doi:10.1038/s41467-023-42696-3)
Supplement: Supplementary file 1 — Supplementary Information [file 41467_2023_42696_MOESM1_ESM.pdf]

Supplementary Information

One-atom-thick boron nitride co-catalyst for enhanced oxygen evolution reactions

Yizhen Lu[1], Bixuan Li[2,3], Na Xu[1], Zhihua Zhou[1], Yu Xiao[1], Yu Jiang[1], Teng Li[1], Sheng Hu[1,4,5], Yongji Gong[2,6*], Yang Cao[1,4,5*]

1 State Key Laboratory of Physical Chemistry of Solid Surfaces, Collaborative Innovation Center of Chemistry for Energy Materials (iChEM), College of Chemistry and Chemical Engineering, Xiamen University, Xiamen 361005, China.
2 School of Materials Science and Engineering, Beihang University, Beijing 100191, China.
3 School of Physics, Beihang University, 100191 Beijing, China.
4 Innovation Laboratory for Sciences and Technologies of Energy Materials of Fujian Province (IKKEM), Xiamen 361005, China.
5 Pen-Tung Sah Institute of Micro-Nano Science and Technology, Xiamen University, Xiamen 361005, China.
6 Tianmushan Laboratory, Hangzhou 310023, China.

*Corresponding authors. Email: yongjigong@buaa.edu.cn; yangcao@xmu.edu.cn

This file concludes:

Supplementary Fig. 1 to 19
Supplementary Table 1 to 4
Supplementary References 1 to 44

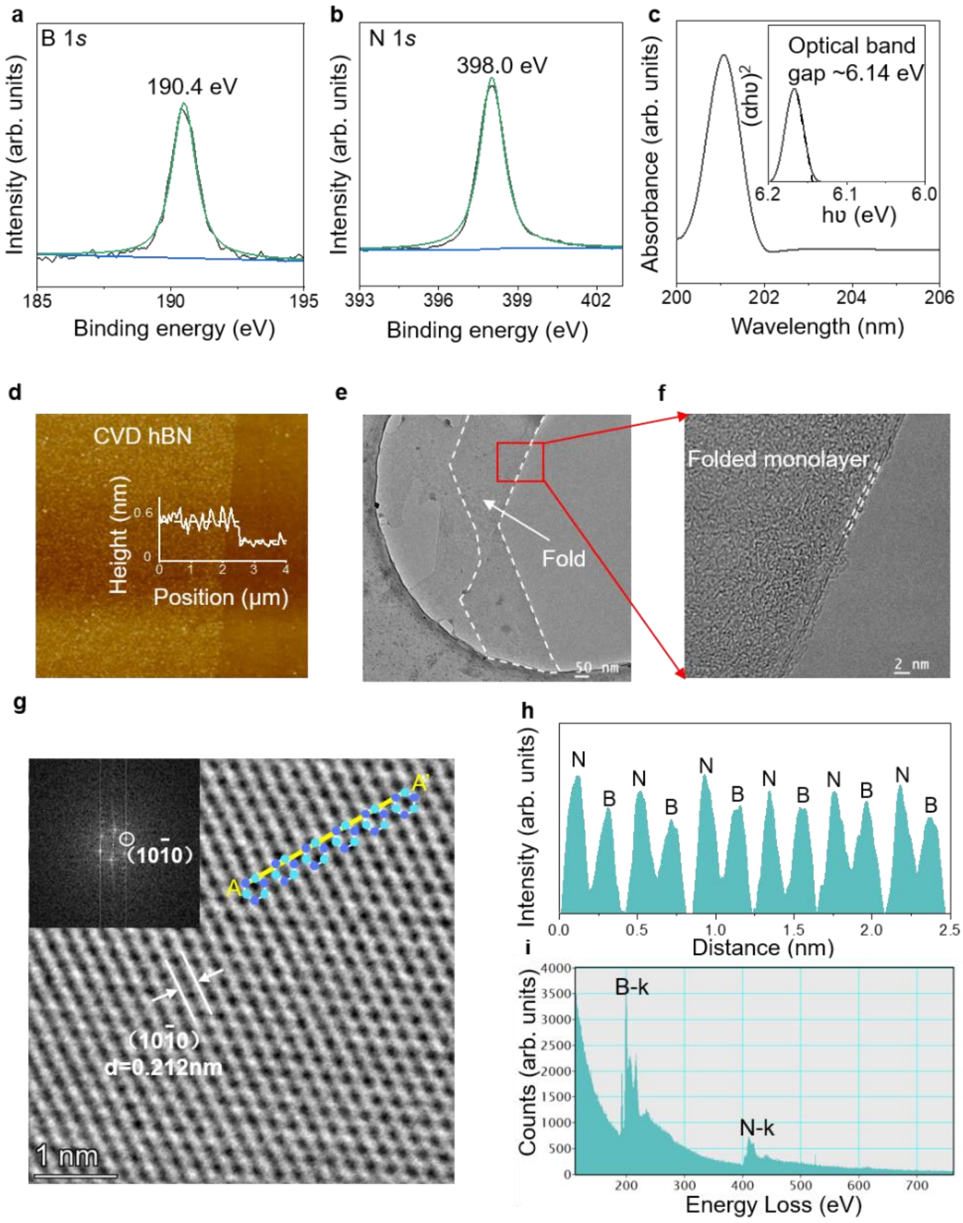

Supplementary Figure 1. Characterization of (hexagonal boron nitride) hBN layers. (a) and **(b)** X-ray photoelectron spectroscopy (XPS) characterizations of as-growth hBN films. The 190.4 eV and 398.0 eV peaks are assigned to B 1*s* and N 1*s* peaks, respectively[1]. **(c)** UV–visible spectra of the hBN film. Inset shows a bandgap of 6.14 eV for our hBN estimated using the data in the figure, which value is consistent with that reported[2]. **(d)** Height profile of a monolayer hBN on silicon substrates measured by atomic force microscope. **(e,f)** High resolution Transmission Electron Microscope images. A bilayer fold is found at the edge of the hBN film, with two monolayers clearly seen. This further proves the monolayer nature of our hBN. **(g)** High Angle Angular Dark Field-Scanning Transmission Electron Microscopy (HAADF-STEM) image of a monolayer hBN crystal. The inset shows the corresponding fast Fourier transform. Cyan and blue balls indicate B and N atoms, respectively. The *d*-spacings of (10$\bar{1}$0) planes of hBN is 0.212 nm. **(h)** Line intensity profile measurements from A-A' in (c) showing the difference in intensity between the B and N atoms. **(i)** Scanning Transmission Electron Microscopy-Electron energy loss spectroscopy (STEM-EELS) spectrum of hBN.

As shown in Supplementary Fig. 1, XPS (Supplementary Fig. 1a, b) analysis prove the B–N chemical bonding structures and UV–visible spectra (Supplementary Fig. 1c) proved that the band gap is consistent with the monolayer hBN reported in the literature[2]. Other characterizations, transmission electron microscope (TEM) and atomic force microscope (AFM) images consistently show that the as-grown hBN is indeed monolayer (Supplementary Fig. 1d-f). The HAADF-STEM (Supplementary Fig. 1g, h) clearly distinguish the B and N atoms in the hexagonal lattice, with higher intensity of N atoms (blue) than that of B atoms (cyan). Fast Fourier transform spots from the whole image (inset of Supplementary Fig. 1g) demonstrate hexagonal spots, assuring the hexagonal structure of the sample. The STEM-EELS spectrum (Supplementary Fig. 1i) also shows two peaks around 198.8 and 410.1 eV, corresponding to K-shell ionization edge of B and N atoms, respectively[1,3]. All these experimental evidences support the monolayer hBN crystal nature of our films.

Ion permeation measurements

To further investigate the impermeability of our CVD hBN crystals, we measured ion transport through the CVD hBN membrane. Schematic of our experimental set-up is shown in Supplementary Fig. 2. The CVD hBN membrane is transferred to a SiN chip with a 2 µm diameter hole, and the SiN chip is mounted in the middle of two reservoirs. Ag/AgCl electrodes are placed inside each reservoir to measure ionic current. No detectable ionic current is obtained within our measurement limit (~5 pA), indicating that the permeability of K^+ and OH^- through the CVD hBN membrane is <10^{-11} S. That indicates a membrane porosity <10^{-6}, which value is consistent with that estimated in gas permeation experiments.

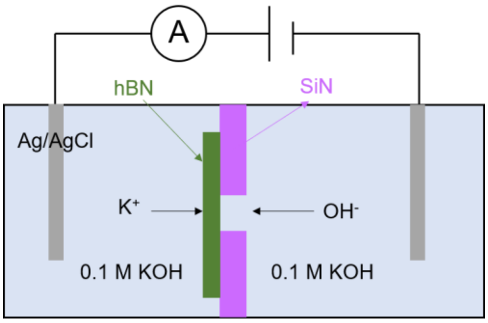

Supplementary Figure 2. Ion permeation measurements set-up.

Gas permeation measurements

To investigate whether our hBN membrane is dense and continuous, we measured helium gas (He) transport through hBN membranes. Schematic of our experimental set-up is shown in Supplementary Fig. 3a. In brief, chemical vapor deposition (CVD) hBN membranes were suspended on an aperture drilled on silicon substrates and were sandwiched between two He leak tight vacuum chambers (leak rate <10^{-14} mol s^{-1}), following the established methods reported in ref. 4. One chamber is filled with helium gas at pressure P_{He} = 1 bar, while the other chamber is kept at vacuum and is connected to a He leak detector (Leybold Quadro Dry). Due to the small kinetic diameter of He gases (kinetic diameter ~2.6 Å), its permeation can be used to detect angstrom-scale defects in membranes[5]. As shown in Supplementary Fig. 3b, however, the permeability of helium through the CVD hBN membrane is under our detection limit (<10^{-14} mol s^{-1}). In a parallel

experiment, we measured the helium permeation through the aperture without hBN coverage, which is about 10^{-8} mol s^{-1}. Within our measurement accuracy limit, we estimate a membrane porosity $<10^{-6}$, or 1 nm^2 defective area per micron meter square.

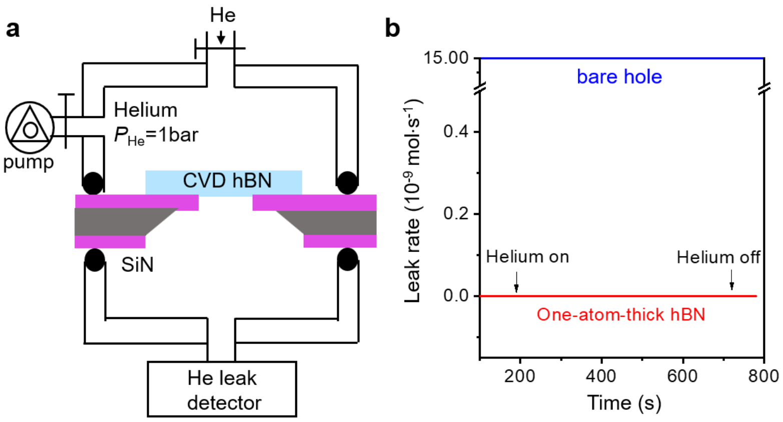

Supplementary Figure 3. Helium transport through hBN membranes. (a) Schematics of gas permeation measurements set-up. Black circles represent rubber O-rings for sealing. **(b)** Leak rate (i.e. He permeation rate) as a function of time. We fill the top chamber with the He gas at the time point marked as "Helium on", and pumped out the He gas at the time point marked as "Helium off".

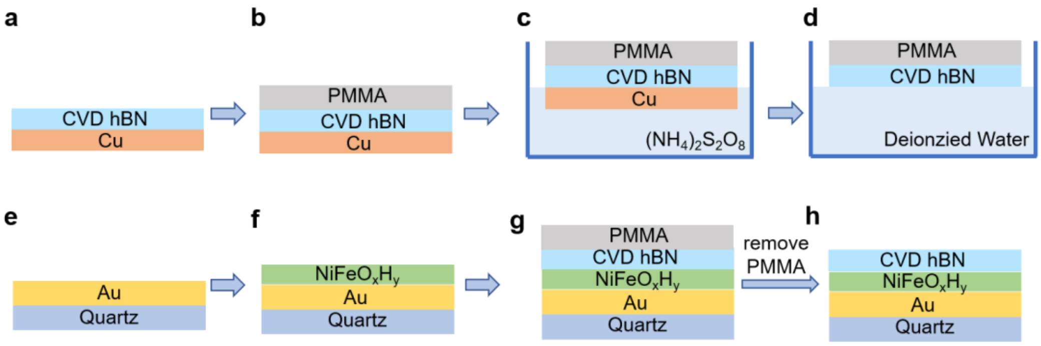

Supplementary Figure 4. Schematic diagram of wet transfer method.

Characterization of $NiFeO_xH_y$ catalytic layer

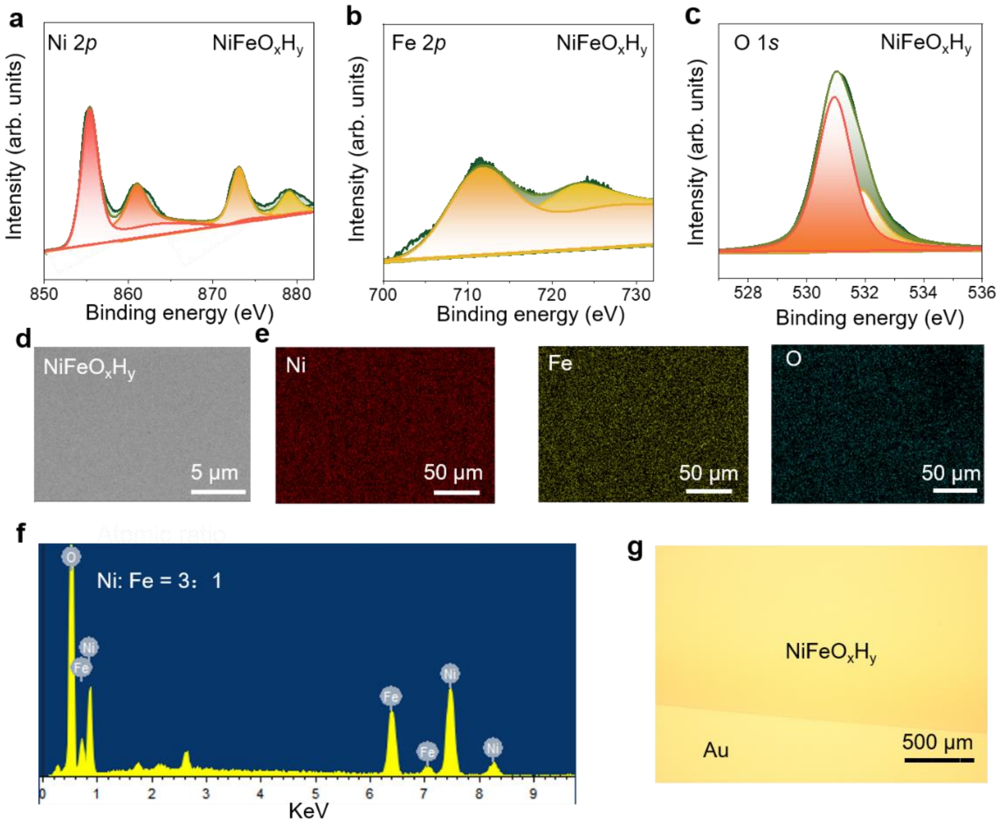

Supplementary Figure 5. Characterization of the electrochemically deposited $NiFeO_xH_y$ catalytic layer. **(a)** to **(c)** X-ray photoelectron spectroscopy of Ni 2*p*, Fe 2*p* and O 1*s* spectrum of $NiFeO_xH_y$, respectively. **(d)** Scanning electron microscope image of the $NiFeO_xH_y$ layer. **(e)** to **(f)** Energy dispersive spectrum for the elemental distribution in the $NiFeO_xH_y$ layer. **(g)** Optical image of $NiFeO_xH_y$ on Au contact. The $NiFeO_xH_y$ layer thickness is about 60 nm.

As shown in Supplementary Fig. 5a to 5c, X-ray photoelectron spectroscopy (XPS) results confirm the presence of Ni, Fe, and O elements. Specifically, the Ni 2*p* spectrum shows two peaks that can be assigned to Ni^{2+}, with Ni $2p_{3/2}$ at 855.4 eV and Ni $2p_{1/2}$ at 873.1 eV. The Fe $2p_{3/2}$ at 711.5 eV can be assigned to Fe^{3+} [6]. The O 1*s* characteristic peak at 531.0 and 531.9 eV can be attributed to lattice oxygen and hydroxy groups[7]. (X-ray Diffraction) XRD characterization shows no diffraction signals, indicating that the $NiFeO_xH_y$ layer is likely to be amorphous. Scanning electron microscopy shows that the obtained $NiFeO_xH_y$ layer is uniform with no visible dis-continuity. Energy dispersive spectrum (EDS) elemental analysis confirms that the Ni, Fe and O elements are uniformly distributed, with a 3:1 atomic ratio between Ni and Fe elements (Supplementary Fig. 5d-f). All these results indicate that the materials prepared are amorphous $NiFeO_xH_y$.

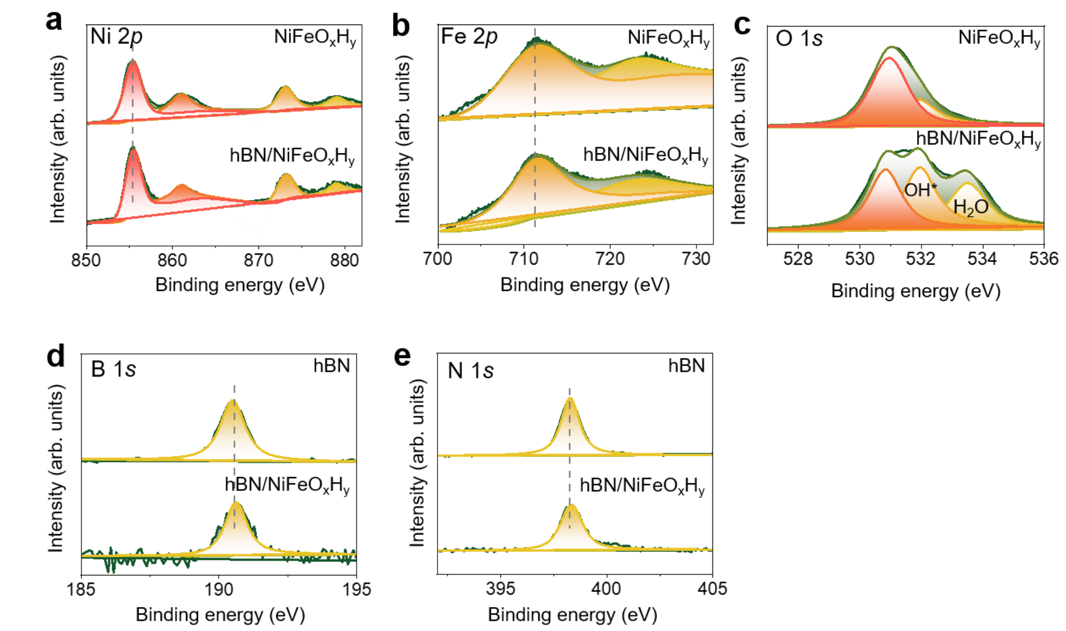

Supplementary Figure 6. X-ray spectrum characterizations of hBN, $NiFeO_xH_y$ and hBN/$NiFeO_xH_y$ heterostructures. (a) Ni 2*p*, **(b)** Fe 2*p* and **(c)** O 1*s* spectrum of $NiFeO_xH_y$ with and without hBN encapsulation. **(d)** B 1*s* and **(e)** N 1*s* spectrum of hBN before and after assembly on $NiFeO_xH_y$ layers. No peak shift is observed in all cases, indicating the absence of bonding between hBN and $NiFeO_xH_y$ layers

The XPS peak attributed to oxygen (O) demonstrates substantial disparity between its profiles before and after hBN encapsulation (Supplementary Fig. 6c). The hBN/$NiFeO_xH_y$ shows three O 1*s* characteristic peaks at 530.9, 531.9, and 533.5 eV. The 530.9 eV peak can be ascribed to lattice oxygen. The peak features positioned at 531.9 and 533.5 eV can be attributed to OH groups and H_2O adsorbed on the surface, respectively[6,7].

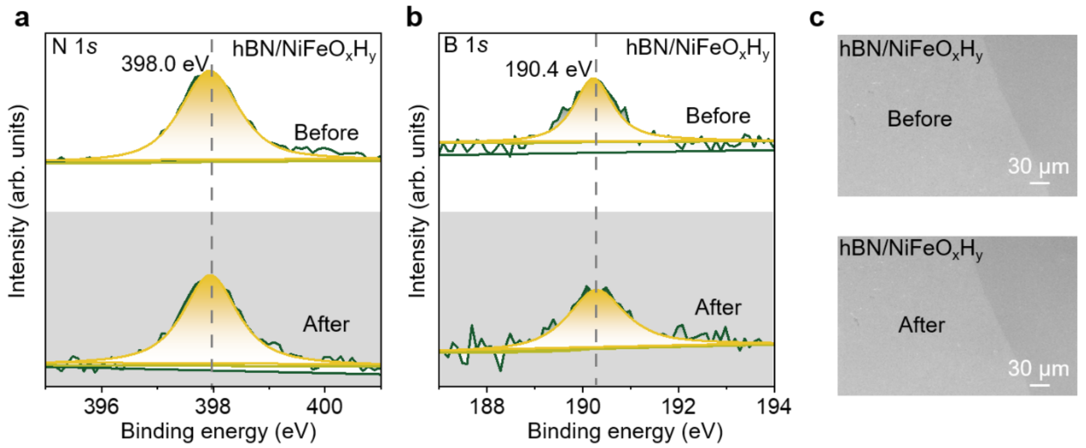

Supplementary Figure 7. Stability of hBN/$NiFeO_xH_y$ heterogeneous electrodes. (a,b) XPS spectra and **(c)** electron microscope characterization of hBN/$NiFeO_xH_y$ before and after OER. No detectable peak shift or visible damage of hBN layer is observed.

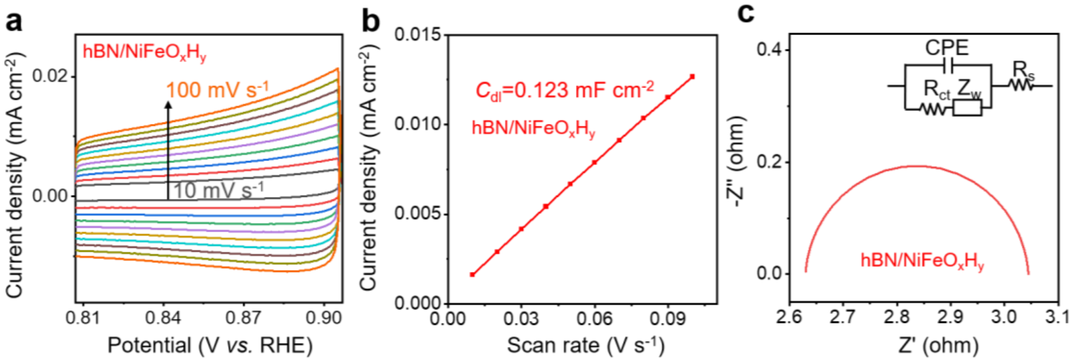

Supplementary Figure 8. The electrochemical active surface area (ECSA) and EIS measurements. **(a)** Cyclic Voltammetry (CV) curves for hBN/$NiFeO_xH_y$ carried out in non-faradic regions at different scan rates in 1M KOH. **(b)** The C_{dl} calculations. **(c)** Electrochemical impedance spectra (EIS) for hBN/$NiFeO_xH_y$.

The ECSA of the hBN/$NiFeO_xH_y$ is 3.08 cm^2, and its J_{ECSA} is 60 mA cm^{-2}@1.53 V which is an order of magnitude higher than other $NiFeO_xH_y$ catalysts in literature[7,8,9].

EIS was measured in 1M KOH (pH = 13.65). In Supplementary Fig. 8c, the Nyquist plot is fitted using Randles equivalent circuit model. The charge transfer resistance of hBN/$NiFeO_xH_y$ catalyst is found to be 0.4 Ω cm^{-2}, which is comparable to that of $NiFeO_xH_y$ reported literatures[10,11]. This is also consistent with our conclusion that the presence of hBN introduces negligible interlayer charge transfer impedance.

Isotope labelling experiments

$NiFeO_xH_y$ were labeled with ^{18}O-isotopes by using $H_2{}^{18}O$ solutions for electrochemically deposition. Afterward, the ^{18}O-labeled catalysts were rinsed with $H_2{}^{16}O$ for serval times to remove the remaining $H_2{}^{18}O$. The Raman peaks of the ^{18}O-labeled hBN/$NiFeO_xH_y$ shifts to lower wavenumbers as compared to that of ^{16}O-labeled hBN/$NiFeO_xH_y$ (Supplementary Fig. 9a), because of the impact of oxygen mass on the vibration mode[12]. This result suggests the successful fabrications of ^{18}O labelled hBN/$NiFeO_xH_y$ samples.

The OER performance of ^{18}O-labeled hBN/$NiFeO_xH_y$ was measured using an analogous method as described in main texts. To analyze their gas products, OER reactions were performed in a closed electrolytic cell at 1.72 V versus RHE. Gas product was transferred from the chamber to our Gas Chromatography-Mass Spectrometry using an injection needle. Signals of three possible products were monitored: $^{16}O^{16}O$, $^{16}O^{18}O$, and $^{18}O^{18}O$. If lattice oxygen oxidation mechanism (LOM) dominate, ^{18}O element is expected to be found in the gas products. However, this expectation is found against our experiments, where no ^{18}O element higher than nature abundance was detected within our detection limit (Supplementary Fig. 9b).

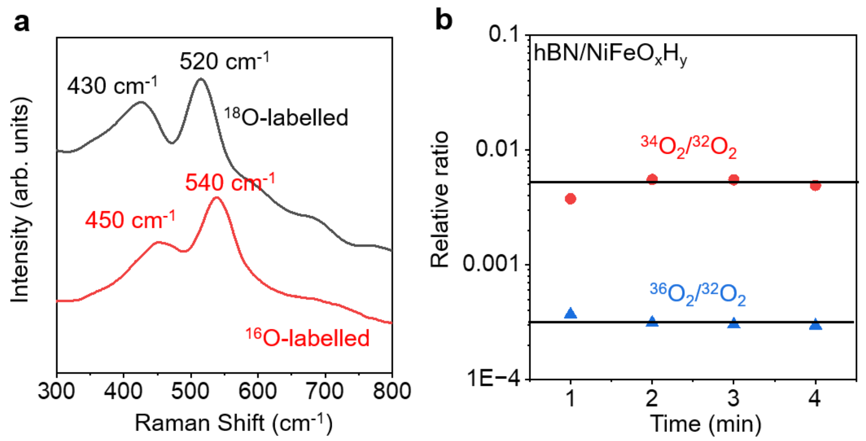

Supplementary Figure 9. ^{18}O isotope labelling experiment. (a) Raman spectrum of ^{18}O-labelled hBN/$NiFeO_xH_y$ electrodes. **(b)** Oxygen products measured by Gas Chromatography-Mass Spectrometry (GC-MS). The relative ratio of $^{36}O_2$ to $^{32}O_2$ and $^{34}O_2$ to $^{32}O_2$ was measured every 60 s. Dots are experimental data. Solid lines represent their natural abundance.

To further prove the encapsulation of our hBN layers, we add tetramethylammonium cation (TMA^+) in the solution and measure the OER performance of hBN/$NiFeO_xH_y$ electrodes. We choose TMA^+ because it is expected to occupy the active sites on $NiFeO_xH_y$ (without hBN coverage) and inhibit OER performance there[13]. In our case of using hBN/$NiFeO_xH_y$ electrodes, however, little change of OER performance was observed. Such result also indicates that our hBN is dense and stable at reaction conditions, preventing any direct contacts between species and the $NiFeO_xH_y$ layer. In addition to results in Supplementary Fig. 9, Supplementary Fig. 10 also indicates that the LOM mechanism is not likely to be the dominant mechanism in our case.

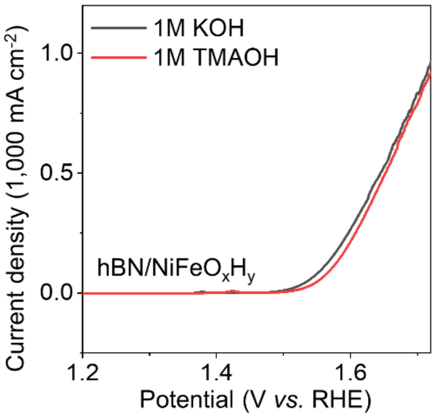

Supplementary Figure 10. Linear sweep voltammetry of hBN/$NiFeO_xH_y$ in KOH and TMAOH.

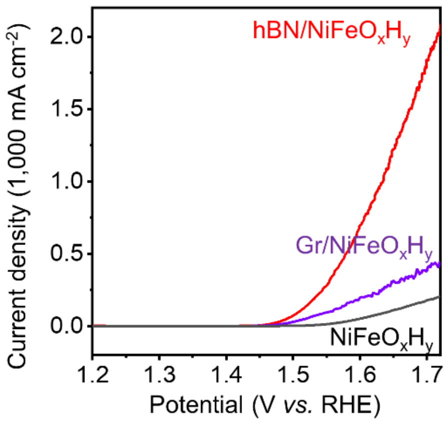

Supplementary Figure 11. Linear sweep voltammetry of hBN/$NiFeO_xH_y$, Graphene (Gr)/$NiFeO_xH_y$ and $NiFeO_xH_y$ electrode. For the preparation of Gr/$NiFeO_xH_y$ electrodes, we use analogous methods to that of preparing hBN/$NiFeO_xH_y$ electrodes, but with the hBN layer being replaced by centimeter sized monolayer CVD graphene.

hBN/$NiFeO_xH_y$ microdevices using mechanically exfoliated monolayer hBN crystals

To fabricate the hBN/$NiFeO_xH_y$ microdevices, first, single layer hBN crystals were mechanically exfoliated onto silicon substrates and transferred onto the $NiFeO_xH_y$/Au contacts using dry transfer techniques[14] (Supplementary Fig. 12 and 13). The lateral size of typical hBN monolayers is about 10 µm. The thickness of hBN monolayers are confirmed by atomic force microscopy (Supplementary Fig. 12). Next, we exposed only the hBN-covered area using e-beam lithography while keeping all other parts of the devices encapsulated with inert polymer. Subsequently, KOH solutions were drop-casted onto the device. A probe station (MPS150, Cascade Microtech) electrically connected the gold electrode with the electrochemical workstation for electrochemistry measurements. Hg/HgO electrode and platinum wires were used as reference electrode and counter electrode, respectively.

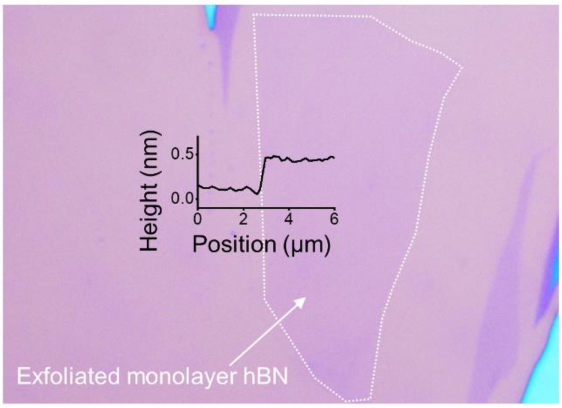

Supplementary Figure 12. Optical image and thickness of the exfoliated monolayer hBN.

As shown in Supplementary Fig. 13, electrodes encapsulated using mechanically exfoliated hBN crystals show a similar OER current density as compared to that from CVD hBN samples, despite the considerably higher defect densities in the latter hBN[15,16]. This result indicates that defects in hBN layers are not likely to play a noticeable role in our case and the basal plane is electro-catalytic.

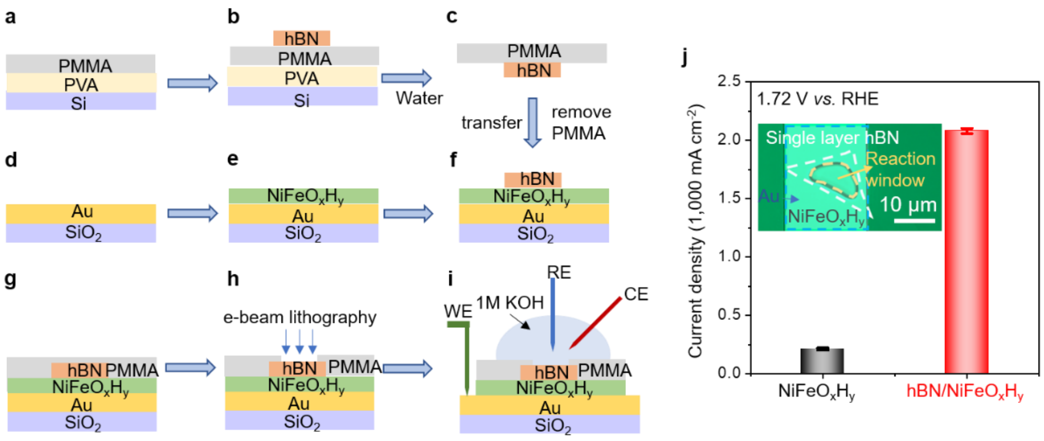

Supplementary Figure 13. Devices encapsulated using mechanically exfoliated monolayer hBN crystals. (a) to **(i)** Schematic of device fabrication and measurement flow using mechanically exfoliated hBN monolayer crystals. PMMA (polymethyl methacrylate) and PVA (polyvinyl alcohol) substrates are used to improve the cleanliness of the obtained hBN crystal[14]. **(j)** Current density diagram of mechanically exfoliated monolayer hBN/$NiFeO_xH_y$ and $NiFeO_xH_y$ samples. Inset shows an optical image of a final device. The area inside the yellow dotted line represents the reaction window defined by e-beam lithography. Error bars represent standard deviations.

Density Functional Theory (DFT) analysis

In our DFT calculations, slab model of the hBN/$NiFeO_xH_y$ heterostructure was built using a Ni/Fe ratio of 3. Structural optimizations were performed by Vienna *Ab-initio* Simulation Package (VASP)[17] with the projector augmented wave (PAW) method[18]. The exchange-functional was treated using the Perdew-Burke-Ernzerhof (PBE) functional[19]. The DFT-D3 correction was introduced to describe the weak interactions between atoms[20]. The cut-off energy of the plane-wave basis was set at 450 eV in structural optimization. For the optimization of both geometry and lattice size, the Brillouin zone integration was performed with 2×2×1 Gamma[21] *k*-point sampling. The self-consistent calculations applied a convergence energy threshold of 10^{-5} eV. The equilibrium geometries and lattice constants were optimized with maximum stress on each atom within 0.02 eV $Å^{-1}$. Hubbard *U* correction was added to describe strong interactions of 3d-orbitals of Fe and Ni, where U_{Fe}=5.3 eV and U_{Ni}=6.6 eV[22,23]. Spin polarization method was adopted to describe the antiferromagnetic hBN/$NiFeO_xH_y$ and $NiFeO_xH_y$. In the structural optimization of hBN/$NiFeO_xH_y$ heterostructure, magnetic moment of half of the Fe and Ni atoms was set at 3μB and 1μB, respectively; magnetic moment of the other half of Fe and Ni atoms was set at -3μB and -1μB, respectively. Initial structure of $NiFeO_xH_y$ and hBN/$NiFeO_xH_y$ is shown in Supplementary Fig. 14.

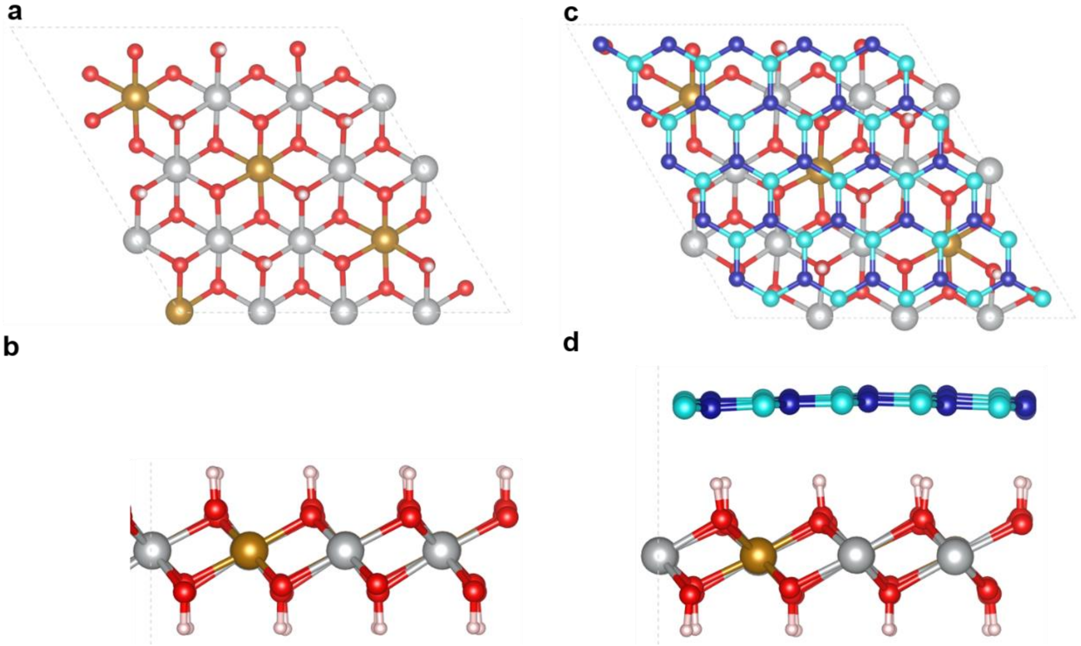

Supplementary Figure 14. (a) and **(b)**, Top and side view of $NiFeO_xH_y$ layer, respectively. **(c)** and **(d)**, top and side view of hBN/$NiFeO_xH_y$ heterostructure, respectively. Fe, Ni, O, B, N and H atoms are indicated by brown, grey, red, cyan, blue and white atoms, respectively.

The isosurface level of charge density difference was set at 0.005 e $Å^{-3}$. Charge density difference was obtained by vaspkit interface[24]. As shown in Supplementary Table 3, we find an interfacial charge transfer of ~0.15 e^- from hBN to $NiFeO_xH_y$ which result indicates the successful creation of the hBN/$NiFeO_xH_y$ heterostructure.

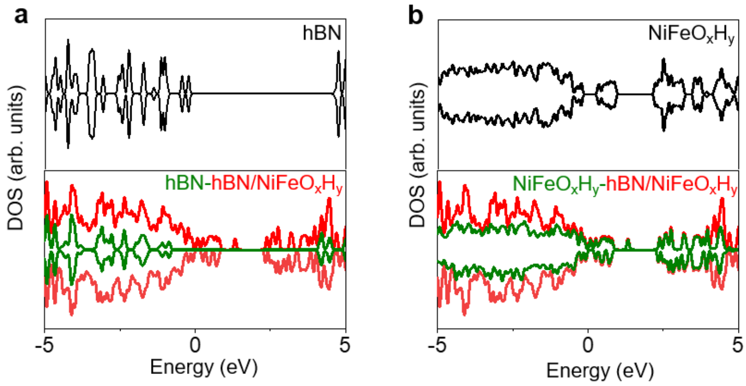

Supplementary Figure 15. Calculated density of states (DOS). (a) Top panel, DOS calculated for individual hBN. Bottom panel, projected DOS of hBN (green line) from the total DOS of hBN/$NiFeO_xH_y$ (red line). **(b)** Top panel, DOS calculated for individual $NiFeO_xH_y$. Bottom panel, projected DOS of $NiFeO_xH_y$ (green line) from the total DOS of hBN/$NiFeO_xH_y$ (red line).

Supplementary Fig. 15 reveals that a reduced bandgap is found for hBN/$NiFeO_xH_y$ heterostructures with respect to that of either hBN or $NiFeO_xH_y$, which result can be attributed to the increased density of states at the Fermi level of $NiFeO_xH_y$ within the heterostructure (Supplementary Fig. 15b). hBN also exhibits a down-shift in its anti-bonding orbitals (Supplementary Fig. 15a) which

leads to a charge transfer of 0.15 electrons (per 1.33 nm^2, which is the area used in our model that contains 25 units of hBN and 16 units of $Ni_{0.75}Fe_{0.25}O_xH_y$) to $NiFeO_xH_y$ (Supplementary Table 3). These results indicate favorable OH* adsorption on hBN surfaces in hBN/$NiFeO_xH_y$ heterostructures. Therefore, we further calculated the OH* adsorption energy and found that, indeed, the adsorption energy on hBN/$NiFeO_xH_y$ (-1.78 eV) is considerably larger than that on bare hBN surface (-0.92 eV) (Supplementary Table 4). Such result further supports the importance of hBN/$NiFeO_xH_y$ heterostructures to OER reactions.

Adsorption energy E_{ads} of OH^- on hBN/$NiFeO_xH_y$ is described as:

$$E_{ads} = E_{t\text{-}OH} - E_t - E_{OH} \quad (1)$$

where $E_{t\text{-}OH}$ is the total energy of OH^- on hBN/$NiFeO_xH_y$. E_t is the total energy of hBN/$NiFeO_xH_y$, without OH^- adsorptions. E_{OH} is the total energy of OH species which are treated as electronically neutral particles in VASP. An analogous expression applies on bare hBN and $NiFeO_xH_y$ layers.

To calculate the Gibbs free energy of OER process, we employ the computational hydrogen electrode (CHE) model developed by Nørskov et al[25]. The elementary steps of oxygen evolution reaction are described as the following[26]:

$$* + H_2O \rightarrow *OH + H^+ + e^- \quad (2)$$

$$*OH \rightarrow *O + H^+ + e^- \quad (3)$$

$$*O + H_2O \rightarrow *O*OH + H^+ + e^- \quad (4)$$

$$*O*OH \rightarrow *O*O + H^+ + e^- \quad (5)$$

$$*O*O \rightarrow * + O_2 \quad (6)$$

where * represents the bare surface of hBN/$NiFeO_xH_y$ heterostructure. *OH, *O and *OOH represents the state of heterostructure surfaces with adsorbed OH, O and OOH intermediates, respectively. Gibbs free energy G of intermediates were calculated as $G = E + E_{zpe} – TS$, where E, E_{zpe} and S indicate energy, zero-point energy and entropy of surface adsorbing H atom, respectively. T is the temperature and T = 298.15 K. The Gibbs free energy of H atom was calculated according to $H_2 \rightarrow H^+ + e^-$, where $G(H^+) = 1/2G(H_2)$. The entropies of the free molecules H_2 and H_2O can be found in the NIST database[27]. Meanwhile, we obtain the free energy of O_2 by the equation of $G(O_2) = 4.92 + 2G(H_2O) - 2G(H_2)$, due to the bad description of magnetism of O_2 in VASP. E_{zpe} and TS of intermediates were obtained by vaspkit interface[24]. pH value was set at 14 to simulate the experimental conditions. The applied potential is set at 0 V as a correction behind ΔG of OER. Thermodynamic activity of OER is judged by examining the rare-limiting potential of the reaction (U_L), which is determined by = - {ΔG_1, ... , ΔG_n}/e, where ΔG_n is the reaction free energy of the nth electrochemical step.

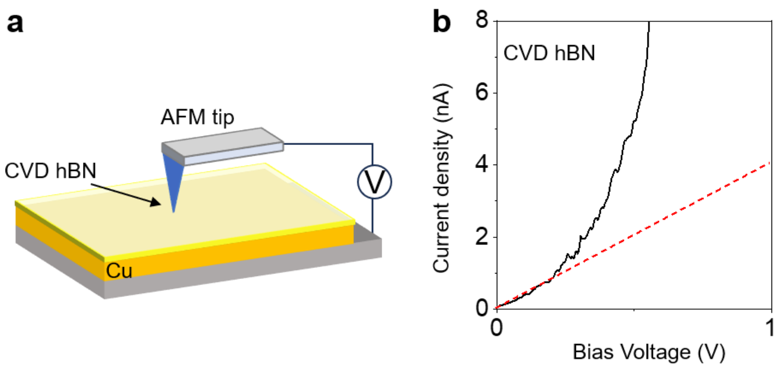

Supplementary Figure 16. hBN conductivity measurements. (a) Schematic diagram of the conductive AFM measurements. **(b)** *I*-*V* curves of single layer hBN on Cu substrate. The red dashed line is the best linear fit using the current at small bias (0 to 200 mV).

To find out the hBN's conductivity along the direction perpendicular to its basal plane, we use conductive AFM technique where the voltage bias is applied between the AFM tip and the copper substrate. The electron conductivity (σ) was calculated with the following equation:

$$\sigma = \frac{1}{\rho} = \frac{1}{R} \times \frac{L}{A} \tag{7}$$

The channel length (L) is the thickness of the single layer hBN, and A is the contact area of AFM tips, where $R = V/I$. ρ is the electrical resistivity. At small bias (10 mV), a tunneling current of 0.1 nA (note that the contact area of the AFM tip is ~2000 nm^2) is found which allows us to estimate an electron conductivity of ~7 x10^{-4} S m^{-1} that is consistent with the values reported in the literature. Further increasing the bias leads to a nonlinear increase of currents which behavior is also a signature of electron tunneling through monolayer hBN crystals[28-30].

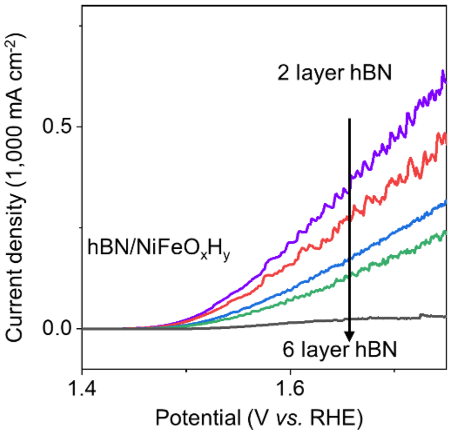

Supplementary Figure 17. Catalytic activity of hBN/$NiFeO_xH_y$ electrodes as a function of the number of hBN films. The multilayer hBN samples are obtained by repeatedly assembly of monolayer hBN films.

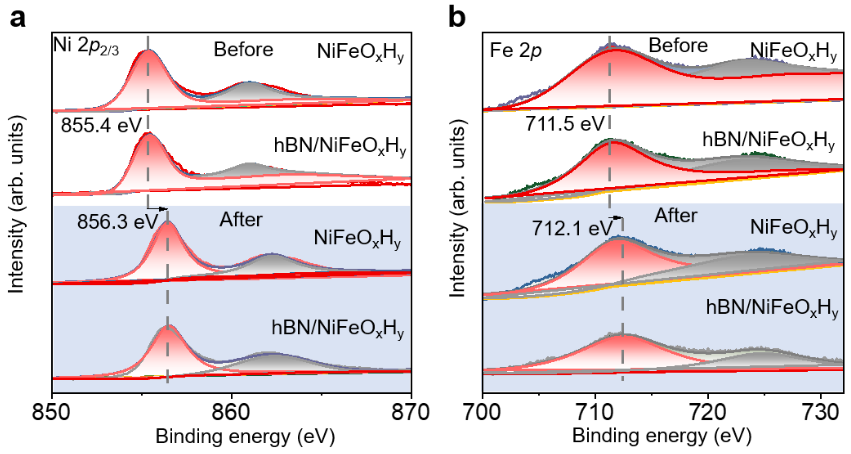

Supplementary Figure 18. XPS characterizations of **(a)** Ni 2$p_{2/3}$ and **(b)** Fe 2p of $NiFeO_xH_y$ and hBN/$NiFeO_xH_y$ before and after electrolysis. The white and blue areas represent before and after the reaction, respectively.

As shown in the Supplementary Fig. 18, the position of both Ni 2$p_{3/2}$ and Fe 2$p_{3/2}$ peaks show similar changes in $NiFeO_xH_y$ and hBN/$NiFeO_xH_y$ cases after the OER reaction. Specifically, the Ni 2$p_{3/2}$ peak shifts by 0.9 eV toward a higher energy, and the Fe 2$p_{3/2}$ peak shifts by 0.6 eV toward a higher energy. This positive shift of binding energy is usually attributed to an electronic state change with a lower electron density and higher oxidation states in Ni and Fe sites, respectively.

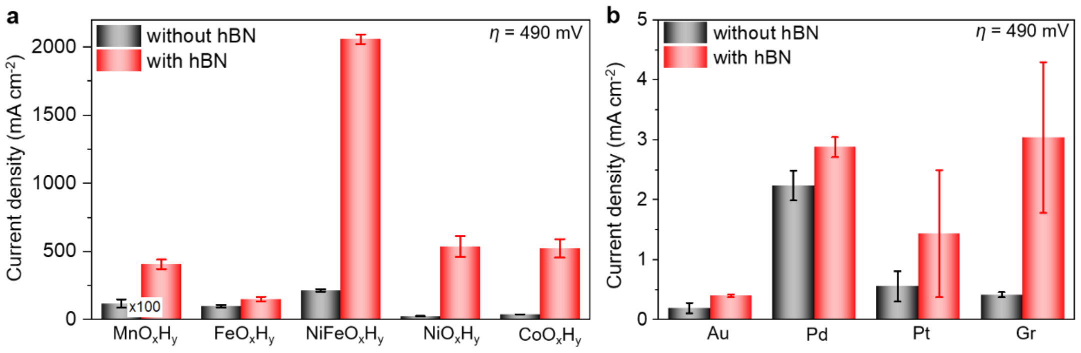

Supplementary Figure 19. Current density diagram of a variety of OER catalysts: **(a)** metal (oxy)hydroxide and **(b)** metal and graphene, with and without hBN cocatalysts. Metal (oxy)hydroxide layers are prepared using electrochemical deposition, following the methods reported previously[31,32]. Metal layers are prepared using e-beam deposition techniques. Graphene monolayers used are from CVD synthesis. Error bars represent standard deviations.

Supplementary Table 1: Comparison of mass activity, overpotential and Tafel slope of hBN/$NiFeO_xH_y$ with other state-of-the-art catalysts.

System	Mass activity ($A\ g^{-1}$)	Overpotential (mV) @100 mA cm^{-2}	Tafel slope (mV dec^{-1})
**hBN/$NiFeO_xH_y$（Our work)**	**40100 @1.53 V**	**279**	**30**
**hBN***	**2.4×10^6 @1.53 V**		
$NiFeO_xH_y$（Our work）	1252 @1.53 V	414	48
Ru-N-C[33]	14284 @1.53 V	340	52.6
MoNiFe-27% (oxy)hydroxide[12]	1910 @1.53 V	290	23
NiFe-27% (oxy)hydroxide[12]	32 @1.53 V	~390	46
RuO_2/C[33]	34.8 @1.53 V	515	62.4
IrO_2NR[34]	2354 @ 1.5 V	240	46.2
Ir-$CaCu_3Ti_4O_{12}$[35]	1195 @1.53 V	~395	44.99
$Co_{0.8}Mn_{0.2}$ MOF-MS 20min[36]	3514.7 @1.5 V	190	78
Cu-Ni-Fe hydr(oxy)oxide[37]	1464.5 @1.53 V	---	44
23% Fe-doped chiral cobalt oxide[38]	1730±178 @1.58 V	---	34
Ni|Fe-[TA]-Catalyst[39]	2900 @1.53 V	---	44.3
WP400[40]	1920 @1.53 V	~360	42.41
NiCoFe-B_i[41]	4320 @1.53 V	---	30.8
$NiFeO_xH_y$-5.4 nm[42]	~8000 @1.53 V	---	---
np-Ir/NiFeO[43]	39300 @ 1.48 V	~240	29.6

*The mass activity of hBN is estimated by extracting the current contribution from hBN divided by its mass. The former one is estimated by subtracting the OER current of $NiFeO_xH_y$ electrodes from that of hBN/$NiFeO_xH_y$ electrodes.

In literatures, various performances of $NiFeO_xH_y$ catalysts have been reported, depending on the specific morphologies of $NiFeO_xH_y$ as well as that of electrode substrates. For example, the high-performance $NiFeO_xH_y$ catalysts usually have highly porous substrates (blank Ni, nickel foam) with a significantly large surface area (around 50-70 cm^2)[7,8]. In fact, in our case, if we normalize the current density by the effective electrochemical active surface area (which is estimated to be 1.22 cm^2), the activity of our devices per unit area is consistent with that reported in literatures. The mass activity of our amorphous $NiFeO_xH_y$ catalyst reaches 1252 A g^{-1}, which is also comparable to many reported $NiFeO_xH_y$ catalysts (Supplementary Table 1).

Supplementary Table 2: Faraday efficiency[44] of $NiFeO_xH_y$ and hBN/$NiFeO_xH_y$ at 1.72 V versus RHE.

	H_2	O_2
$NiFeO_xH_y$	0.93	0.93
hBN/$NiFeO_xH_y$	0.99	1.00

Supplementary Table 3. Calculated charge density of $hBN/NiFeO_xH_y$ heterostructure.

	The number of atoms at the corresponding site	Average charge at each site (e^-)	The total charge of each material (e^-)
$hBN/NiFeO_xH_y$ Ni site	12	1.2644	-0.15
$hBN/NiFeO_xH_y$ Fe site	4	1.7162	
$hBN/NiFeO_xH_y$ O site	32	-0.9979	
$hBN/NiFeO_xH_y$ H site	16	0.6093	
$hBN/NiFeO_xH_y$ B site	25	2.130	0.15
$hBN/NiFeO_xH_y$ N site	25	-2.124	

Supplementary Table 4. OH* adsorption energy of hBN, $NiFeO_xH_y$ and $hBN/NiFeO_xH_y$.

Material	OH* adsorption energy (eV)
hBN	-0.92
$NiFeO_xH_y$	-0.59
$hBN/NiFeO_xH_y$	-1.78

Supplementary References

1. Fukamachi, S. et al. Large-area synthesis and transfer of multilayer hexagonal boron nitride for enhanced graphene device arrays. *Nat. Electron.* **6**, 126–136 (2023).
2. Elias, C. et al. Direct band-gap crossover in epitaxial monolayer boron nitride. *Nat. Commun.* **10**, 2639 (2019).
3. Yu, C. et al. Defect-Induced Efficient Heteroepitaxial Growth of Single-Wall Carbon Nanotubes @ Hexagonal Boron Nitride Films. *Materials* **16**, 1864 (2023).
4. Zhou, Z. et al. Gas permeation through graphdiyne-based nanoporous membranes. *Nat. Commun.* **13,** 4031 (2022).
5. Bunch, J. S. et al. Impermeable Atomic Membranes from Graphene Sheets. *Nano Lett.* **8**, 2458–2462 (2008).
6. Bao, F. et al. Host, Suppressor, and Promoter—The Roles of Ni and Fe on Oxygen Evolution Reaction Activity and Stability of NiFe Alloy Thin Films in Alkaline Media. *ACS Catal.* **11**, 10537–10552 (2021).
7. Lan, B.-L. et al. Metal-Organic Frameworks-Derived Nickel–Iron Oxyhydroxide with Highly Active Edge Sites for Electrochemical Oxygen Evolution. *Small Struct.* **3**, 2200085 (2022).
8. Yang, H. et al. Preparation of nickel-iron hydroxides by microorganism corrosion for efficient oxygen evolution. *Nat. Commun.* **11**, 5075 (2020).
9. Zhong, B., Kuang, P., Wang, L. & Yu, J. Hierarchical porous nickel supported $NiFeO_xH_y$ nanosheets for efficient and robust oxygen evolution electrocatalyst under industrial condition. *Appl. Catal. B* **299**, 120668 (2021).
10. Liu, D. et al. Heat-Triggered Ferri-to-Paramagnetic Transition Accelerates Redox Couple-Mediated Electrocatalytic Water Oxidation. *Adv. Funct. Mater.* **32**, 2111234 (2022).
11. Li, P. et al. Tuning Electronic Structure of NiFe Layered Double Hydroxides with Vanadium Doping toward High Efficient Electrocatalytic Water Oxidation. *Adv. Energy Mater.* **8**, 1703341 (2018).
12. He, Z. et al. Activating lattice oxygen in NiFe-based (oxy)hydroxide for water electrolysis. *Nat. Commun.* **13**, 2191 (2022).

13. Wang, X. et al. Pivotal role of reversible NiO_6 geometric conversion in oxygen evolution. *Nature* **611**, 702–708 (2022).
14. Dean, C. R. et al. Boron nitride substrates for high-quality graphene electronics. *Nat. Nanotechnol.* **5**, 722–726 (2010).
15. Roy, S. et al. Structure, Properties and Applications of Two-Dimensional Hexagonal Boron Nitride. *Adv. Mater.* **33**, 2101589 (2021).
16. Shanmugam, V. et al. A Review of the Synthesis, Properties, and Applications of 2D Materials. *Part. Part. Syst. Charact.* **39**, 2200031 (2022).
17. Hafner, J. Ab-initio simulations of materials using VASP: Density-functional theory and beyond. *J Comput Chem* **29**, 2044-2078 (2008).
18. Blöchl, P. E. Projector augmented-wave method. *Phys. Rev. B* **50**, 17953-17979 (1994).
19. Perdew, J. P., Burke, K. & Ernzerhof, M. Generalized Gradient Approximation Made Simple. *Phys. Rev. Lett.* **77**, 1396 (1997).
20. Grimme, S. Semiempirical GGA-type density functional constructed with a long-range dispersion correction. *J Comput Chem* **27**, 1787-1799 (2006).
21. Monkhorst, H. J. & Pack, J. D. Special points for Brillouin-zone integrations. *Phys. Rev. B* **13**, 5188-5192 (1976).
22. Anisimov, V. I., Zaanen, J. & Andersen, O. K. Band theory and Mott insulators: Hubbard U instead of Stoner I. *Phys. Rev. B* **44**, 943-954 (1991).
23. Anisimov, V. I., Aryasetiawan, F. & Lichtenstein, A. I. First-principles calculations of the electronic structure and spectra of strongly correlated systems: the LDA+ U method. *J. Phys.: Condens. Matter* **9**, 767–808 (1997).
24. Wang, V., Xu, N., Liu, J.-C., Tang, G. & Geng, W.-T. VASPKIT: A User-friendly Interface Facilitating High-throughput Computing and Analysis Using VASP Code. *Comput. Phys. Commun.* **267**, 108033 (2021).
25. Skúlason, E. et al. Density functional theory calculations for the hydrogen evolution reaction in an electrochemical double layer on the Pt (111) electrode. *Phys. Chem. Chem. Phys.* **9**, 3241-3250 (2007).
26. Jing, Y., Zhou, Z., Geng, W., Zhu, X. & Heine, T. 2D Honeycomb-Kagome Polymer Tandem as Effective Metal-Free Photocatalysts for Water Splitting. *Adv. Mater.* **33**, 2008645 (2021).
27. NIST Computational Chemistry Comparison and Benchmark Database, NIST Standard Reference Database Number 101, Release 21, August 2020, edited by Russell D. Johnson, III, Available at http://cccbdb.nist.gov/.
28. Lee, G.-H. et al. Electron tunneling through atomically flat and ultrathin hexagonal boron nitride. *Appl. Phys. Lett.* **99**, 243114 (2011).
29. Summerfield, A. et al. Moiré-Modulated Conductance of Hexagonal Boron Nitride Tunnel Barriers. *Nano Lett.* **18**, 4241–4246 (2018).
30. Wang, P. et al. Scalable Synthesis of Monolayer Hexagonal Boron Nitride on Graphene with Giant Bandgap Renormalization. *Adv. Mater.* **34**, 2201387 (2022).
31. Taitt, B. J., Nam, D.-H. & Choi, K.-S. A Comparative Study of Nickel, Cobalt, and Iron Oxyhydroxide Anodes for the Electrochemical Oxidation of 5-Hydroxymethylfurfural to 2,5-Furandicarboxylic Acid. *ACS Catal.* **9**, 660–670 (2019).
32. Burke, M. S., Kast, M. G., Trotochaud, L., Smith, A. M. & Boettcher, S. W. Cobalt−Iron (Oxy)hydroxide Oxygen Evolution Electrocatalysts: The Role of Structure and Composition on

Activity, Stability, and Mechanism. *J. Am. Chem. Soc.* **137**, 3638–3648 (2015).
33. Cao, L. et al. Dynamic oxygen adsorption on single-atomic Ruthenium catalyst with high performance for acidic oxygen evolution reaction. *Nat. Commun.* **10**, 4849 (2019).
34. Liao, F. et al. Iridium oxide nanoribbons with metastable monoclinic phase for highly efficient electrocatalytic oxygen evolution. *Nat. Commun.* **14,** 1248 (2023).
35. Thao, N. T. T. et al. Colossal Dielectric Perovskites of Calcium Copper Titanate ($CaCu_3Ti_4O_{12}$) with Low-Iridium Dopants Enables Ultrahigh Mass Activity for the Acidic Oxygen Evolution Reaction. *Adv. Sci.* **10**, 2207695 (2023).
36. Zhou, G. et al. Spin-state reconfiguration induced by alternating magnetic field for efficient oxygen evolution reaction. *Nat. Commun.* **12**, 4827 (2021).
37. Cai, Z. et al. Amorphous Nanocages of Cu-Ni-Fe Hydr(oxy)oxide Prepared by Photocorrosion For Highly Efficient Oxygen Evolution. *Angew. Chem. Int. Ed.* **58**, 4189-4194 (2019).
38. Vadakkayil, A. et al. Chiral electrocatalysts eclipse water splitting metrics through spin control. *Nat. Commun.* **14**, 1067 (2023).
39. Hou, S. et al. Metamorphosis of Heterostructured Surface-Mounted Metal-Organic Frameworks Yielding Record Oxygen Evolution Mass Activities. *Adv. Mater.* **33,** 2103218 (2021).
40. Kim, Y. J. et al. Highly efficient oxygen evolution reaction via facile bubble transport realized by three-dimensionally stack-printed catalysts. *Nat. Commun.* **11**, 4921 (2020).
41. Feng, C. et al. A self-healing catalyst for electrocatalytic and photoelectrochemical oxygen evolution in highly alkaline conditions. *Nat. Commun.* **12**, 5980 (2021).
42. Roy, C. et al. Impact of nanoparticle size and lattice oxygen on water oxidation on $NiFeO_xH_y$. *Nat. Catal.* **1,** 820-829 (2018).
43. Jiang, K. et al. Dynamic active-site generation of atomic iridium stabilized on nanoporous metal phosphides for water oxidation. *Nat. Commun.* **11**, 2701 (2020).
44. Kempler, P. A. & Nielander, A. C. Reliable reporting of Faradaic efficiencies for electrocatalysis research. *Nat. Commun.* **14**, 1158 (2023).
